# Integrating Genome-Wide Association and eQTLs Studies Identifies the Genes and Gene Sets Associated with Diabetes

**DOI:** 10.1155/2017/1758636

**Published:** 2017-06-28

**Authors:** Xiao Liang, Awen He, Wenyu Wang, Li Liu, Yanan Du, Qianrui Fan, Ping Li, Yan Wen, Jingcan Hao, Xiong Guo, Feng Zhang

**Affiliations:** Key Laboratory of Trace Elements and Endemic Diseases of National Health and Family Planning Commission, School of Public Health, Health Science Center, Xi'an Jiaotong University, Xi'an, China

## Abstract

**Aim:**

To identify novel candidate genes and gene sets for diabetes.

**Methods:**

We performed an integrative analysis of genome-wide association studies (GWAS) and expression quantitative trait loci (eQTLs) data for diabetes. Summary data was driven from a large-scale GWAS of diabetes, totally involving 58,070 individuals. eQTLs dataset included 923,021 cis-eQTL for 14,329 genes and 4,732 trans-eQTL for 2,612 genes. Integrative analysis of GWAS and eQTLs data was conducted by summary data-based Mendelian randomization (SMR). To identify the gene sets associated with diabetes, the SMR single gene analysis results were further subjected to gene set enrichment analysis (GSEA). A total of 13,311 annotated gene sets were analyzed in this study.

**Results:**

SMR analysis identified 6 genes significantly associated with fasting glucose, such as C11ORF10 (*p value* = 6.04 × 10^−8^), MRPL33 (*p value* = 1.24 × 10^−7^), and FADS1 (*p value* = 2.39 × 10^−7^). Gene set analysis identified HUANG_FOXA2_TARGETS_UP (false discovery rate = 0.047) associated with fasting glucose.

**Conclusion:**

Our study provides novel clues for clarifying the genetic mechanism of diabetes. This study also illustrated the good performance of SMR approach and extended it to gene set association analysis for complex diseases.

## 1. Introduction

Diabetes is a group of metabolic diseases, mainly characterized by raised blood glucose over a prolonged period. Without effective treatments, diabetes will lead to serious secondary disorders, such as heart disease, stroke, chronic kidney failure, and foot ulcers. During the past decades, the prevalence of diabetes continues to increase, caused by aging, obesity, smoking, and other unhealthy lifestyle factors [1]. It was estimated that 334 million individuals would suffer diabetes in 2025 [1]. Diabetes has become one of the major public health problems, bringing heavy economic burden to the society.

Genetic factors contribute greatly to the development of diabetes. Extensive genetic studies have been conducted and identified a group of susceptibility genes for diabetes, such as PTEN [2], SREBF1 [3], JAZF1 [4], BCL2 [5], and FAM19A2 [5]. However, the genetic risk of diabetes explained by the identified loci was limited, suggesting the existence of undiscovered susceptibility loci for diabetes. The missing heritability can partly be attributed to the regulatory genetic variants, which are mostly locating outside genes and ignored by traditional genetic studies.

Expression quantitative trait loci (eQTLs) are a group of important regulatory loci, which can regulate gene expression levels. The disease-associated SNPs identified by GWAS are significantly enriched in eQTLs, supporting the implication of eQTLs in the pathogenesis of complex diseases [6]. Through genome-wide detecting associations between gene transcript abundance and genomic polymorphisms, a large amount of eQTLs has been identified in human genome [7, 8]. Recently, summary data-based Mendelian randomization (SMR) analysis was proposed to utilize extensive published GWAS as well as eQTLs data. SMR is capable of integrating GWAS summary and eQTLs annotation data to identify novel causal genes, the expression levels of which are associated with target diseases [9]. SMR showed a high power for identifying novel causal genes of complex diseases [9].

In this study, we conducted a genome-wide single gene and gene sets expression association analysis for diabetes. SMR was first applied to a large-scale GWAS data for screening novel susceptibility genes of diabetes. To gain insight into the biological significance of identified genes, we extended SMR to gene set enrichment analysis (GSEA). SMR gene-level analysis results were subjected to GSEA for identifying diabetes associated gene sets with known functional information.

## 2. Methods

### 2.1. GWAS Summary Datasets

A large-scale GWAS meta-analysis summary data of diabetes was used in this study [10]. Briefly, this GWAS comprised 58,070 individuals from 29 studies involved in the Meta-Analysis of Glucose and Insulin related traits Consortium. Fasting glucose and fasting insulin were measured from whole blood, plasma, or serum samples. Detailed information of measurements of fasting glucose and fasting insulin is summarized in Supplementary Table S1 and Table S2 in Supplementary Material available online at https://doi.org/10.1155/2017/1758636. Commercial platforms were used for genome-wide SNP genotyping, such as Affymetrix 500K SNP array, Illumina 550K, and Perlegen 600K. Imputation was conducted by MACH [11] or IMPUTE [12] against the HapMap CEU reference genome (build 36). The GWAS meta-analysis was conducted by joint meta-analytical approach [13]. Detailed information of cohorts, genotyping, imputation, meta-analysis, and quality control approaches can be found in the published studies [10].

### 2.2. SMR Single Gene Analysis

The GWAS meta-analysis summary data of diabetes was input into SMR for single gene expression association analysis of fasting glucose and insulin resistance. SMR is capable of integrating GWAS results with eQTLs annotation information to evaluate the relationships between gene expression levels and complex traits [9]. We applied the eQTLs annotation dataset built by Westra et al. [14]. Briefly, these eQTLs datasets were driven from a meta-analysis of 5,311 peripheral blood samples and replicated in another 2,775 samples. Illumina whole-genome Expression BeadChips were used for gene expression profiling. SNP genotyping was conducted using commercial platforms, such as Illumina 610K quad arrays and Illumina HumanHap300 arrays. Imputation was conducted using MACH [11] or IMPUTE [12] against the HapMap 2 reference panels. 923,021 cis-eQTL for 14,329 gene expression probes and 4,732 trans-eQTL for 2,612 gene expression probes were identified at false discovery rate (FDR) < 0.05 [14]. An expression association testing *p* value for each gene was calculated by SMR. After Bonferroni correction, the genes with SMR *p* values < 9.28 × 10^−6^ (0.05/5389) were considered as significant genes in our study.

### 2.3. Gene Set Enrichment Analysis

To reveal the functional significance of identified genes, the SMR single gene expression association testing results were further subjected to GSEA [15]. The gene set annotation database (msigdb.v5.1) was obtained from the GSEA Molecular Signatures Database (http://software.broadinstitute.org/gsea/msigdb/index.jsp). 5,000 permutations were conducted to calculate the FDR adjusted *p value* of each gene set [16]. Significant gene sets were identified at FDR adjusted *p value* < 0.05. Detailed GSEA procedures can be found in our previous studies [17].

## 3. Results

### 3.1. SMR Single Gene Expression Association Analysis

A total of 5,389 genes with both GWAS summary and eQTLs data were analyzed in this study. After strict Bonferroni correction, SMR identified 6 genes significantly associated with fasting glucose (Table 1), including C11ORF10 (*p* value = 6.04 × 10^−8^), MRPL33 (*p* value = 1.24 × 10^−7^), FADS1 (*p value *= 2.39 × 10^−7^), ACP2 (*p* value = 1.74 × 10^−6^), NR1H3 (*p value *= 1.78 × 10^−6^), and SNX17 (*p* value = 2.19 × 10^−6^).

For fasting insulin, SMR detected suggestive association signals for 7 genes (Table 2), including ATRIP (*p value *= 9.68 × 10^−5^), MRPL33 (*p value *= 9.75 × 10^−6^), ATRIP (*p value *= 1.90 × 10^−4^), POLR1E (*p value *= 2.60 × 10^−4^), AMT (*p value *= 3.44 × 10^−4^), TNFSF13 (*p value *= 4.55 × 10^−4^), and POLR1E (*p value *= 7.82 × 10^−4^).

### 3.2. Gene Set Enrichment Analysis

A total of 10,987 annotated gene sets were analyzed in this study. GSEA observed significant association between HUANG_FOXA2_TARGETS_UP gene ontology (GO) term and fasting glucose (FDR adjusted *p value* = 0.047). For fasting insulin, GSEA detected suggestive association signal for chr8p23 GO term (FDR adjusted *p value* = 0.063).

## 4. Discussion

It is a challenge to reveal the biological significances of identified loci by GWAS, especially a large part of significant loci locating outside genes [9]. To better understand the genetic basis and make full use of published GWAS data of diabetes, we conducted an eQTL-based single gene and gene set expression association analysis for diabetes. We identified multiple genes and gene sets associated with fasting glucose or fasting insulin.

SMR analysis observed the most significant association between fasting glucose and C11ORF10. C11ORF10 is close to another significant gene FADS1 identified by SMR. It has been demonstrated that C11ORF10 played an important role in fatty acid and glucose metabolism [18]. Zabaneh and Balding reported that C11ORF10 and FADS1 were significantly associated with metabolic syndrome [19]. Powell et al. observed that FADS1 knockout mice presented less glucose and insulin excursions during oral glucose tolerance tests along with lower fasting glucose, insulin, triglyceride, and total cholesterol levels [20]. Yao et al. suggested that FADS1-FADS2 gene cluster was significantly associated with type 2 diabetes [21]. Cormier et al. observed that FADS gene cluster could modulate plasma fasting glucose and fasting insulin levels in response to n-3 polyunsaturated fatty acids supplementation [22].

SNX17 is another notable gene associated with fasting glucose. SNX17 encodes sorting nexin 17, which involves receptor binding and phosphatidylinositol binding. It has been demonstrated that the eQTLs of SNX17 was significantly associated with glucometabolic phenotypes [23]. Adachi and Tsujimoto found that SNX17 directly interacted with FEEL-1/stabilin-1, which was implicated in the development of diabetes [24].

TNFSF13 is significantly associated with fasting insulin in this study. Gao et al. reported that the TNFSF13 level in serum was significantly associated with the diabetic status of patients with pancreatic ductal adenocarcinoma-associated diabetes [25].

Besides confirming functional relevance of previously reported candidate genes with diabetes, SMR analysis also identified several novel candidate genes for diabetes, such as MRPL33, ACP2, and NR1H3. To the best of our knowledge, few efforts have been paid to investigate the potential roles of these genes in the development of diabetes. Further biological studies are warranted to confirm our finding and clarify the potential roles of novel candidate genes in the pathogenesis of diabetes.

Gene set analysis found that HUANG_FOXA2_TARGETS_UP GO term was significantly associated with fasting glucose. HUANG_FOXA2_TARGETS_UP comprises 45 genes, some of which have been suggested to be implicated in the development of diabetes, such as KAT2B and TNFAIP3. Rabhi et al. found that disruption of KAT2B led to impaired insulin secretion and glucose intolerance in mice [26]. They suggested that KAT2B was a key transcriptional regulator in maintaining normal function of adaptive *β* cell [26]. TNFAIP3 was suggested to be associated with type 1 diabetes [27].

In summary, we conducted a genome-wide integrative analysis of GWAS and eQTLs data for diabetes. We identified several novel candidate genes and gene sets associated with the risk of diabetes. Our results provide new clues for clarifying the genetic mechanism of diabetes. We also illustrated the good performance of SMR approach and extended it to gene set association analysis for complex diseases.

## Supplementary Material

Table S1: The details of analysis metrics and methods of fast glucose for all cohorts.
Table S2: The details of analysis metrics and methods of fasting insulin for all cohorts.

## Figures and Tables

**Table 1:** List of candidate genes identified by SMR for fasting glucose.

Gene	Top SNP	MAF	SMR	
			*β*	*p* value
C11ORF10	rs174547	0.331	−0.059	6.04 × 10^−8^
MRPL33	rs3736594	0.258	−0.118	1.24 × 10^−7^
FADS1	rs174548	0.301	−0.067	2.39 × 10^−7^
ACP2	rs901746	0.297	−0.050	1.74 × 10^−6^
NR1H3	rs901746	0.297	−0.051	1.78 × 10^−6^
SNX17	rs1260320	0.392	−0.072	2.19 × 10^−6^

*Note*. MAF, minor allele frequency.

**Table 2:** List of candidate genes identified by SMR for fasting insulin.

Gene	Top SNP	MAF	SMR	
			*β*	*p* value
ATRIP	rs2228561	0.129	−0.070	9.68 × 10^−5^
MRPL33	rs3736594	0.258	−0.067	9.75 × 10^−5^
ATRIP	rs2228561	0.129	−0.084	1.90 × 10^−4^
POLR1E	rs10758435	0.166	−0.026	2.60 × 10^−4^
AMT	rs1050088	0.429	0.031	3.44 × 10^−4^
TNFSF13	rs9898876	0.193	−0.037	4.55 × 10^−4^
POLR1E	rs10973396	0.168	−0.028	7.82 × 10^−4^

*Note*. MAF, minor allele frequency.
