# Supplementary material for: Integrating Genome-Wide Association and eQTLs Studies Identifies the Genes and Gene Sets Associated with Diabetes
Source: Biomed Res Int. 2017 Jun 28;2017:1758636. doi: 10.1155/2017/1758636 (PMC5506468; doi:10.1155/2017/1758636)
Supplement: Supplementary file 1 — Table S1: The details of analysis metrics and methods of fast glucose for all cohorts. Table S2: The details of analysis metrics and methods of fasting insulin for all cohorts. [file 1758636.f1.docx]

**Table S1:** Measurements of fast glucose for each cohort

| Cohort | Country | Sample | Collecting method | Assay |
| --- | --- | --- | --- | --- |
| CHS | USA | 12-hr fasting serum | Venipuncture was performed on study participants under 12-hour fasting conditions | Kodak Ektachem 700 Analyzer |
| FHS | USA | Fasting plasma | ≥8 hr overnight fast | Hexokinase reagent kit (agent glucose test, AbbottSouth Pasadena, California) |
| DGI | Finland,  Sweden | Fasting plasma and blood | 12 hour overnight fast | Glucose oxidase method (Beckman Glucose Analyzer, Beckman Instruments, Fullerton, CA) and glucose dehydrogenase method (Hemocue,Angelholm, Sweden) |
| BLSA | USA | Fasting plasma | Overnight fast | ELISA (Alpco Diagnostic) |
| FUSION | Finland | Fasting plasma | Overnight fast and plasma collected in EDTA tubes | Glucose oxidase method (Yellow Springs instruments, Yellow Springs, OH and autoanalyser) and hexokinase method |
| CoLaus | Switzerland | Fasting venous fresh plasma | Centrifuged and analyzed within 2 hours | Glucose dehydrogenase (Roche Diagnostics, CH) |
| InCHIANTI | Italy | Fasting venous fresh plasma | _ | Enzymatic colorimetric assay using a modified glucose oxidase-peroxidase method (Roche Diagnostics GmbH, Mannheim, Germany) |
| NFBC1966 | Finland | Fasting blood | Blood collected between 0800 and  1100 h. Sample mixed with EDTA anticoagulant and precipitated with 0.5ml of perchloric acid | Glucose dehydrogenase method (granutest 250, Diagnostica Merck, Darmstadt, Germany) |
| NTR/ NESDA | Netherlands | Fasting plasma | Overnight fast, glucose measured or samples snapfrozen within 5 hr of collection | Vitros 250 glucose assay (Johnson & Johnson, Rochester, USA) |
| Rotterdam Study | Netherlands | Fasting plasma | Serum separated by centrifugation and quickly frozen in liquid nitrogen | Glucose hexokinase method |
| PROCARDIS | UK,  Sweden, Italy, Germany | Fasting plasma | Overnight fast | Hexokinase/glucose-6-phosphate dehydrogenase method on a Hitachi 917 autoanalyzer (Roche Diagnostics, Mannheim, Germany) |
| Sorbs | Germany | 75g OGTT(fasting, 30 min,120 min), serum | Overnight fast, spinning within 1 hour after collection, then immediate quick-freeze on dry ice before transport, further storage in -80°C freezer | Hexokinase method (Automated analyser Modular, Roche Diagnostics, Mannheim, Germany). |
| ERF | Netherlands | Serum | Overnight fast, morning venepuncture, serum separated and frozen immediately | Hexokinase on Synchron LX20 |
| CROAS  (Vis Study) | Croatia | Serum (gel separated) | Fasting blood drawn in serum gel tube, between 8 and 9am. Sample allowed to clot for 30min then centrifuged and serum aliquoted then stored at-70C until analysis. | UV hexokinase photometry |
| ORCADES (Orkney) | Scotland | Serum (gel separated) | Fast from 10pm prev night, blood drawn in serum gel tube, between 8-9.30 am, taken to biochem lab w / in 2h | HK / G6PDH method (Synchron GLU) (Beckman Coulter) |
| MICROS (Tyrol) | Italy | plasma | Fast from prev night, blood without anticoagulant, between 7-9.30 am, taken to biochem lab w / in 3h | Hexokinase method (Dimension RxL Dade Behring instrumentation) |
| AGES | Iceland | fasting serum | Venous, ≥8 hr overnight fast | Measured on a Hitachi 912 using reagents from Roche Diagnostics, glucose-oxidase method |
| ARIC | USA | Fasting serum | Venous, ≥8 hr overnight fast | Hexokinase assay on a Coulter DACOS (Coulter Instruments); interassay analytical SD was 1.3 mg / dL (CV, 1.6%) at 79.3 mg / dL |
| BSN | Fenland | Fasting plasma | Venous | HITACHI 917 |
| FamHS | US | Fasting serum | Venous | Glucose-oxidase (Kodak Ektachem 700 Analyzer, Rochester, NY) |
| Fenland | UK | Fasting venous fresh plasma with fluoride | Plasma centrifuged immediately and analyzed same day (within 4h) | Hexokinase / glucose-6- phosphate dehydrogenase (Dimension RxL, Siemens) |
| French Adult Controls | France | Fasting plasma | Morning after overnight fast | Glucose oxidase colorimetric assay using a modified glucose oxidase-peroxidase method |
| GENOA | USA | Fasting blood sample | Blood samples were drawn after over-night fast | Hexokinase reagent from Boehringer Mannheim (Indianapolis, IN 46256) on a Hitachi 911 Chemistry Analyzer (Roche Diagnostics, Indianapolis, IN) for serum |
| GenomEUtwin | Sweden, Denmark, Finland, The Netherlands | Fasting plasma | Venous+ | NED: Vitros 250 Glucose, Johnson&Johnson, Rochester, USA; DEN; FIN; SWE |
| HABC | United States | Fasting serum | Venous, ≥8 hr overnight fast | Vitros Glucose; Ortho-Diagnostics, Johnson&Johnson; Rochester, NY USA |
| Health  2000 | Finland | Fasting serum | ≥4 h of fasting | Hexokinase, enzymatic, Merck Diagnostica |
| Korcula | Croatia | Serum (gel separated) | Fasting blood drawn in serum gel tube, between 8 and 9am. Sample allowed to clot for 30min then centrifuged and serum aliquoted then stored at -70C until analysis. | UV hexokinase photometry |
| Split | Croatia | Serum (gel separated) | Fasting blood drawn in serum gel tube, between 8 and 9am. Sample allowed to clot for 30min then centrifuged and serum aliquoted then stored at -70C until analysis. | UV hexokinase photometry |
| SUVIMAX | France | Fasting venous fresh plasma | Collected in recumbent participants who were fasting for 12-h overnight | Enzymatic assay using the hexokinase method (Advia 1650, Bayer Diagnostics, Puteaux, France) |

**Table S2:** Measurements of fasting insulin for each cohort

| Cohort | Country | | | Sample | Collection method | Assay |
| --- | --- | --- | --- | --- | --- | --- |
| CHS | USA | | | Fasting serum | Venipuncture was performed on study participants under 12-hour fasting conditions | Kodak Ektachem 700 Analyzer |
| FHS | USA | | | Fasting plasma | ≥8 hr overnight fast | DPC Coat-A-Count RIA(total immunoreactive insulin) |
| DGI | Finland,  Sweden | | | Serum | 12 hour overnight fast | Radioimmunoassay(Pharmacia, Uppsala,Sweden), enzyme linked immunoassay (DAKO  Diagnostics Ltd, Cambridgeshire,UK), fluoroimmunometric assay (AutoDelfia, Perkin Elmer Finland, Turku, Finland) |
| BLSA | USA | | | Fasting plasma | Overnight fast | Glucose analyzer (Beckman Instruments) |
| FUSION | Finland | | | Fasting plasma(FUSION and serum (Finrisk02) | _ | RIA with dextran charcoal separation |
| CoLaus | Switzerland | | | Fasting frozen venous plasma | Fasting venous blood was immediatelly centrifuged and plasma frozen at -80C until measurement | Solid-phase, two-site chemiluminescentimmunometric assay (Diagnostic Products, Los Angeles, USA) |
| InCHIANTI | | | Italy | Fasting venous fresh plasma | Centrifuged and analyzed within 2 hours | Double-antibody, solid-phase radioimmunoassay (SorinBiomedica, Milan, Italy) |
| NFBC1966 | | Finland | | Fasting Serum | Overnight fast | RIA( Pharmacia Diagnostics, Uppsala,Sweden) |
| NTR / NESDA | | Netherlands | | Fasting heparin plasma | Overnight fast, Insulin measured or samples snapfrozen within 5 hr of collection | Immulite 1000 Insulin(Siemens Medical Solutions) |
| Rotterdam Study | | Netherlands | | Fasting blood | Overnight fast | Modular analytics E170 and cobas e 601 analyzers |
| PROCARDIS | | UK, Sweden, Italy, Germany | | Fasting plasma | Overnight fast | ELISA (DAKO Ltd, Cambridgeshire, UK) |
| Sorbs | | Germany | | 75g OGTT (fasting, 30 min,120 min), serum | Overnight fasting, spinning within 1 hour after collection, then immediate quick-freeze on dry ice before transport, further storage in -80°C freezer | AutoDELFIA Insulin assay (PerkinElmer Life and Analytical Sciences, Turku, Finland). |
| ERF | | Netherlands | | Serum | Overnight fast, morning venepuncture, serum separated and frozen immediately | INS-Irma kit (Biosource) |
| CROAS  (Vis Study) | | Croatia | | Serum (gel separated) | Fasting blood drawn in serum gel tube, between 8 and 9am. Sample allowed to clot for 30min then centrifuged and serum aliquoted then stored at-70C until analysis. | Electrochemiluminescenceimmunoassay ECL1A(Cobas) |
| ORCADES (Orkney) | | Scotland | | Serum (gel separated) | Fast from 10pm prev night, blood drawn in serum gel tube, between 8-9.30 am, taken to biochem lab w/ in 2h | Access Ultrasensitive insulin (immuno-enzymatic method) (Beckman Coulter) |
| MICROS (Tyrol) | | Italy | | _ | _ | _ |
| AGES | | Iceland | | fasting serum | Venous, ≥8 hr overnight fast | Measured by an electrochemiluminescenceimmunoasssay on a Roche Elecsys 2010 instrument, using two monoclonal antibodies and a sandwich principleAccess Ultrasensitive insulin (immuno-enzymatic method) (Beckman Coulter) |
| ARIC | | USA | | fasting serum | Venous, ≥8 hr overnight fast | Radioimmunoassay (Cambridge Biomedical); interassay analytical SD was 2.3 mU / L (CV, 17%) at 13.5 mU / L |
| BSN | | Fenland | | Fasting plasma | Venous | ELISA - TOSOH |
| FamHS | | US | | Fasting serum | Venous | Radioimmunoassay (Coat-A--Count, Diagnostic Products Corporation, Los Angeles, CA) |
| Fenland | | UK | | Fasting venous plasma with heparin | Fasting venous blood was immediatelly centrifuged and plasma frozen at -80C until measurement | 1235 AutoDELFIA automatic immunoassay system using a two-step time resolved fluorometric assay Kit No. B080-101 (Perkin Elmer) |
| French Adult Controls | | France | | Fasting plasma | Morning after overnight fast | Micro particle enzyme inmmunoassay |
| GENOA | | USA | | Fasting blood sample | Blood samples were drawn after over-night fast | Two-site immunoenzymatic assay performed on the Access automated immunoassay system (Beckman Instruments, Chaska, MN) |
| GenomEUtwin | | Sweden, Denmark, Finland, The Netherlands | | Fasting plasma | Venous | NED: Immulite Insulin, DPC, Los Angeles, USA; DEN: ; FIN: ; SWE: |
| HABC | | United States | | Fasting serum | Venous, ≥8 hr overnight fast | Microparticle Enzyme Immunoassay (MEIA) on the Abbot Imx; Abbott Laboratories Diagnostics Division, South Pasadena, CA |
| Health  2000 | | Finland | | Fasting serum | ≥4 h of fasting | Microparticle immunoassay, Abbott Laboratories, Dainabot, Tokyo Japan |
| Korcula | | Croatia | | Serum (gel separated) | Fast from 10pm prev night, blood drawn inserum gel tube, between 8-9.30 am, taken to biochem lab w / in 2h | Access Ultrasensitive insulin (immuno-enzymatic method) (Beckman Coulter) |
|  | |  | |  |  |  |
| Split | | Croatia | |  |  |  |
| SUVIMAX | | France | | _ | _ | _ |
